# Management of massive retropubic haematoma post-TVT

**DOI:** 10.1007/s00192-016-3025-5

**Published:** 2016-05-14

**Authors:** Lucy May, Swati Jha, Shahram Abdi

**Affiliations:** Department of Urogynaecology, Jessop Wing, Sheffield Teaching Hospitals, Sheffield, S10 2SF UK

**Keywords:** TVT, Retropubic haematoma

## Introduction

**Fig. 1:**
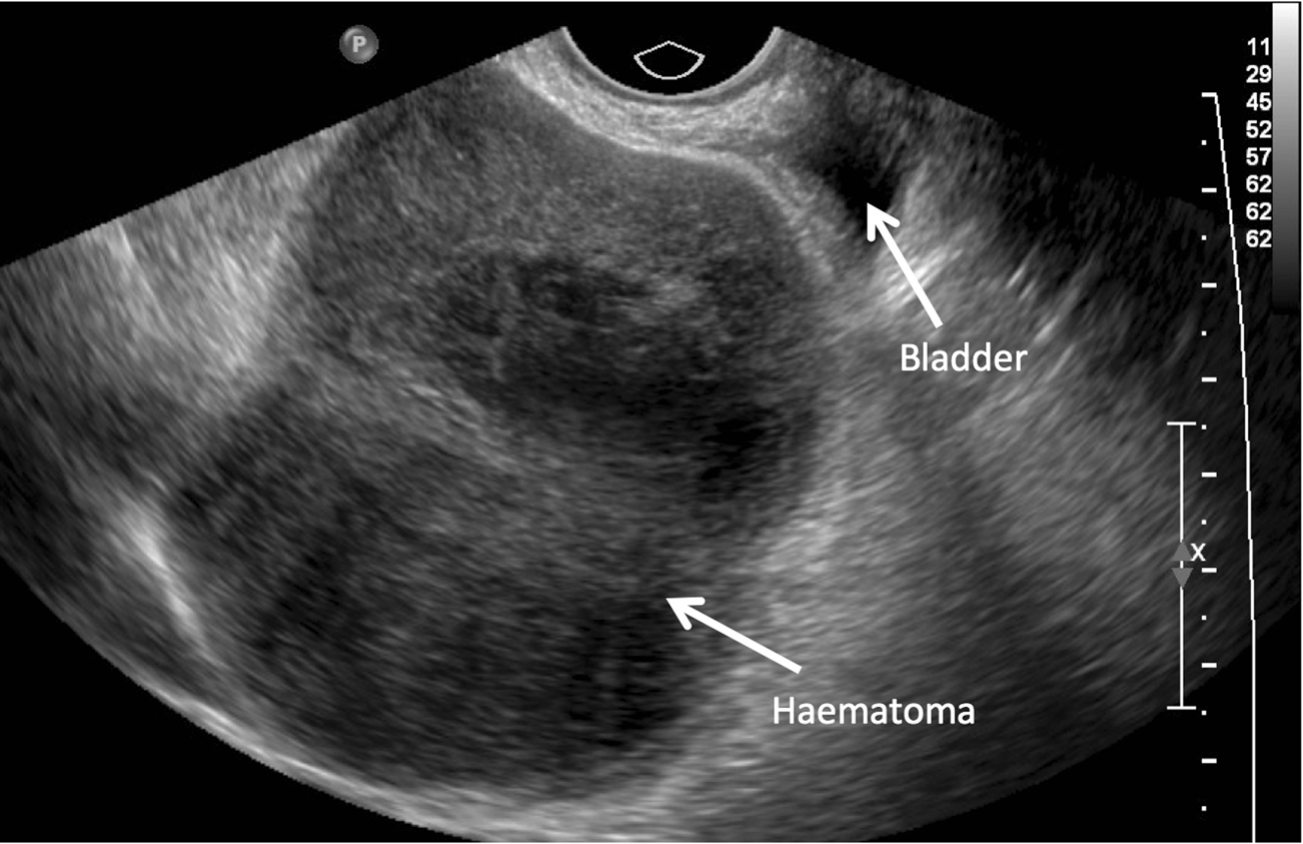
Predrainage transvaginal ultrasound scan of haematoma = 9 cm (ultrasound depth 8.1 cm)

**Fig. 2:**
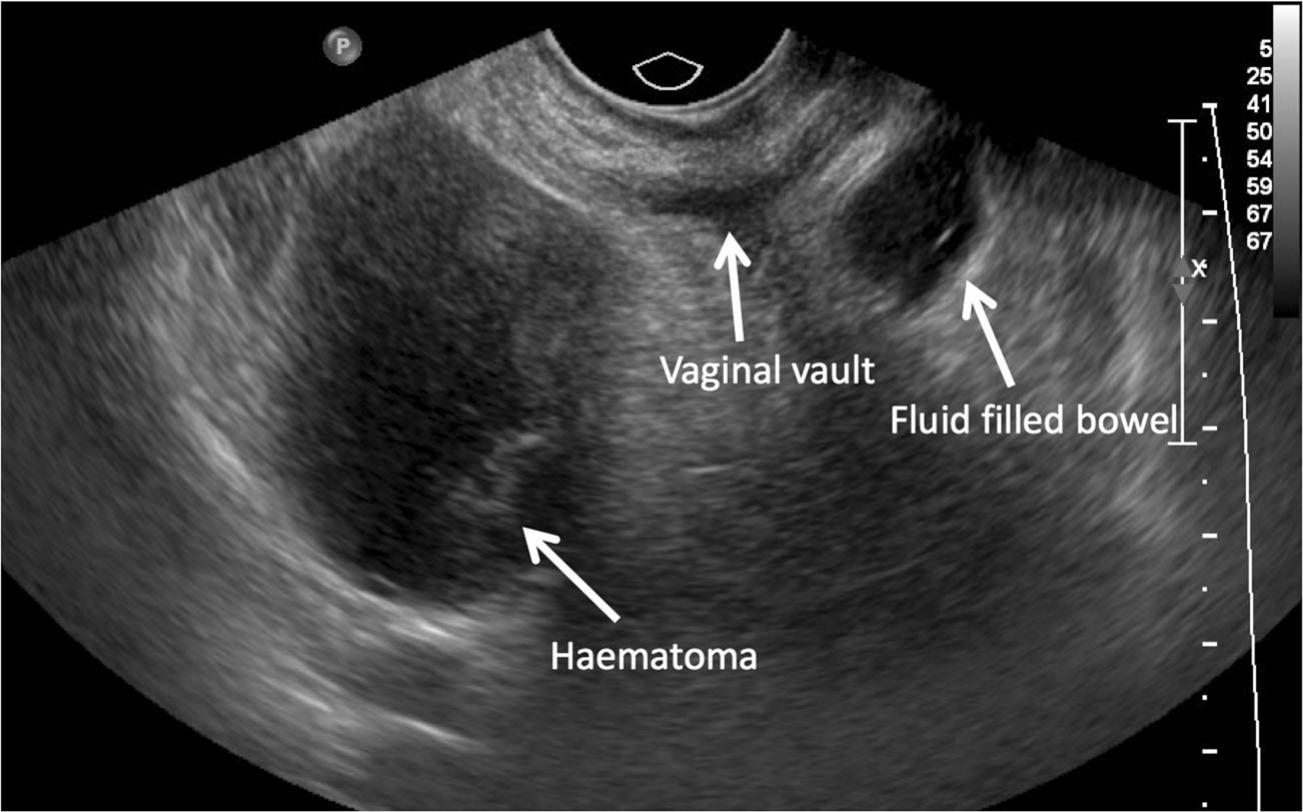
Predrainage transvaginal ultrasound scan of haematoma = 5 cm (ultrasound depth 7.1 cm, making haematoma more magnified)

Tension-free vaginal tape (TVT) is well established for the treatment of stress urinary incontinence (SUI), with a cure rate of 84 % [1]. A known complication is the formation of a retropubic haematoma in 1.5–3 % [1] of cases. A massive haematoma is one >8 cm and/or a 4 gm % drop in haemoglobin. Management is often surgical intervention via a laparotomy or observation until spontaneous resolution (Figs 1 and 2).

## Case Report

A 74-year-old patient underwent TVT for SUI. She was discharged home the next day but presented with pain, urgency and persistent urinary leakage 1 week later. On review, the wound was healing normally but an ultrasound (US) scan revealed a 9-cm organising haematoma. She underwent US-guided drainage of the haematoma, and a size 12-F locking drain was inserted and left on free drainage for 48 h; this was flushed twice daily to prevent blockage. On day 2, the drain was removed; 200 ml of blood was drained. Repeat US 4 weeks later showed the collection to be halved, with a diameter of 5 cm and complete symptom resolution (Figs 1 and 2).

Although not a common complication after a TVT, massive retropubic haematomas can be life threatening due to significant blood loss. A review of the literature demonstrates surgical drainage via a laparotomy in most cases. One study identified 2,091 patients over a 15-year period who underwent a midurethral sling procedure, seven of whom developed a haematoma, with six requiring surgical intervention either by laparotomy, vaginal drainage or suprapubic drainage [2]. Retziusscopy has also been reported as a management technique for haematomas after a TVT [3].

This case demonstrates that US-guided drainage can be an effective treatment for massive retropubic haematomas in stable patients, providing good recovery of bladder function. It avoids repeat anaesthetic and further surgery and therefore is a safe approach.
